# Complete molecular spectrum of β-globin gene mutations via direct sequencing identifies seven novel variants in β-thalassemia major

**DOI:** 10.1371/journal.pone.0336610

**Published:** 2025-11-07

**Authors:** Torin Abdulaziz Sadoon, Hemin Esmael Othman

**Affiliations:** 1 Department of Medical Laboratory Technology, College of Health and Medical Technology-Shekhan, Duhok Polytechnic University, Kurdistan Region, Iraq; 2 Department of Biology, College of Science, University of Duhok, Kurdistan Region, Iraq; Shaheed Rajaei Cardiovascular Medical and Research Center: Rajaie Cardiovascular Medical and Research Center, IRAN, ISLAMIC REPUBLIC OF

## Abstract

**Background:**

A common monogenic condition, β-thalassemia, is caused by a variety of mutations in the β-globin (*HBB*) gene. It is essential to accurately characterize these mutations for genetic counselling, diagnosis, and treatment.

**Objective:**

This study aimed to characterize and provide an updated and complete molecular spectrum of β-globin gene mutations, including both known and novel mutations, estimate their frequencies, and determine their possible deleterious effects.

**Methods:**

A total of 60 major β-thalassemia patients who sought treatment at Jeen Hospital in Duhok from August 2024 to February 2025 were analyzed using direct DNA sequencing of the β-globin gene.

**Results:**

Out of 60 sequence chromatograms, 40 were of excellent quality. Among these, 26 distinct mutations were found, comprising 10 exonic and 16 intronic variants. The most prevalent benign variants were IVSII-16 G > C (80%) and IVS II-666 C > T (77.5%), followed by Cd2 T > C [HBB:c.9 T > C, (62.5%)]. The pathogenic exonic mutations were found in coding regions, including Cd5 -CT [HBB:c.17_18delCT, (17.5%)], Cd6 A > T [HBB:c.20A > T (sickle cell mutation, 2.5%)], Cd8 A > G [HBB:c.26A > G (2.5%)], Cd39 C > T [HBB:c.118C > T, (5%)], Cd44 C > T [HBB:c.134C > T, (2.5%)], and Cd44 -C [HBB:c.135delC, (2.5%)]. Pathogenic intronic variants were also documented in splice junctions, including IVS I-1 G > A (15%) and IVS I-5 G > C (17.5%). Notably, seven novel variants were detected in this study, including four intronic variants (IVS I-129 + C Ins, IVS II-72 G > A, IVS II-579 G > A, and IVS II-763 + C Ins) and three exonic variants [Cd44 C > T (HBB:c.135C > T), Cd47 –G (HBB:c.142delG), and Cd118 –TT (HBB:c.355_356delTT)]. The majority of which were expected to be pathogenic or likely pathogenic based on variant location, predicted functional effect, and observed frequencies.

**Conclusion:**

The molecular investigation of β-thalassemia patients in Duhok showed a significant level of genetic variability in the β-globin gene and a high prevalence of compound heterozygosity among the β-thalassemia patients. The finding of several new variants is significant since it adds to the current mutation database and broadens the known mutational spectrum of the β-globin gene in this community. It also supports the necessity of thorough molecular diagnostics in regional management, screening, and genetic counseling of β-thalassemia.

## Introduction

β-thalassemia is a widespread anemia of a monogenic hereditary disorder with an autosomal recessive pattern of inheritance that occurs due to mutations in the beta-globin gene (*HBB*) [1]. The degree of β-globin chain imbalance is determined by the nature of the mutation of the β-globin gene. β0 refers to the complete absence of β-globin from the affected allele. β+ refers to alleles with some residual production of β-globin (around 10%). In β++, the reduction in β-globin production is very mild.

Clinically, β-thalassemia is linked to splenomegaly and bone marrow hyperplasia in addition to severe anemia [2]. At the clinical level, β-thalassemia can be classified into three primary categories: minor β-thalassemia (β/β+ or β/0), β-thalassemia intermedia (β0/β+), and β-thalassemia major (β0/β0) [3]. Two beta-globin gene abnormalities are the source of β-thalassemia major, which results in severe anemia and associated problems. Individuals identified as having β-thalassemia major require ongoing blood transfusions along with other medical interventions like iron chelating medications to prevent iron overload [4].

The human β-globin locus consists of five functional genes, *HBE*, *HBG2*, *HBG1*, *HBD*, and *HBB*, positioned on chromosome 11 in the same order in which they are expressed during ontogenesis. The β-Globin gene is found in the β-globin locus on the short arm of chromosome 11(11p15.4) and is composed of five genes (β-globin and beta-like genes span 70 kb). The β-Globin gene spans 1,600 bp, which encodes 146 amino acid residues. It consists of three exons and two introns (intervening sequences, IVS). Exon 1 encodes for amino acid residues from 1 to 29 and also encodes the first two bases of codon 30. Exon 2 encodes the last base of codon 30 and amino acids from 31 to 104. Exon 3 codes for amino acids from 105 to 146. In addition, 5’ flanking, promoter, splicing, and 3’ untranslated (3’-UTR) regions are also important for β-globin protein activity [5].

Presently, approximately 400 mutations in the beta-globin gene have been reported worldwide; the majority of them are single-nucleotide substitutions that result in faulty expression of the β-globin gene and lead to the disease’s broad genotypic and phenotypic variability. Rarely, β-thalassemia results from gross gene deletion [6]. Although a handful of mutations constitute the bulk of beta-thalassemia mutations in each particular population, the distribution and frequency of mutations vary among ethnic groups and geographical locations [7]. Furthermore, studies are reporting new and uncommon mutations, suggesting that all regions need to be characterized for mutations, and the information about these mutations should be made public for prenatal diagnostics.

The World Health Organization estimated that approximately 68,000 people are born with β-thalassemia each year, and the incidence of β-thalassemia is estimated to be one in 100,000 worldwide [8]. High incidence has been reported in the Mediterranean, the Middle East, the Indian subcontinent, and Southeast Asia [3]. The high frequency of β-thalassemia among the Middle East population, particularly the Kurdish community, is thought to be largely caused by the traditional customs of consanguineous marriage and marriage within the same caste or ethnicity. With an average heterozygous carrier incidence of 4% and an estimated 15,000 registered individuals with thalassemia major and intermedia, β-thalassemia is a significant health issue in Iraq. With significant geographical differences in the occurrence of β-thalassemia mutations, hemoglobinopathies are serious health issues among Iraqi Kurds, with carrier rates ranging from 3.7% to 6.9% in the Kurdistan Region, Iraq. Of these, 27 distinct β-thalassemia mutations have been documented, based on published literature in this region of the world. The issue is made worse by the approximately 30% consanguineous marriage rate [7]. In this case, it would be imperative to start a preventive program for this illness. Establishing a program like this in a given group requires first identifying the mutations causing β-thalassemia in that population and building a database about the pattern and frequency of beta-globin mutations. Additionally, improved disease prevention would be made possible by identifying the uncommon and unique β-thalassemia variations.

Several studies have partially addressed the molecular basis of β-thalassemia in Duhok city, based on conventional methods of polymerase chain reaction and reverse hybridization of oligonucleotide probes corresponding to known β-thalassemia mutations [9]. However, aside from a single case-study report [10], to the best of our knowledge, the literature contains no previous research using direct DNA sequencing and mutation analysis of the whole β-globin gene. This would result in a knowledge deficit in the investigation of unexplored β-globin gene sites, as uncommon or new mutations are frequently missed by conventional techniques, which may lead to an incomplete understanding of the disease disorders, complicating diagnosis and treatment options. Thus, the current study aimed to provide a comprehensive molecular spectrum of β-globin gene mutations, define the unusual and/or novel mutations of hitherto unexplored sites of the β-globin gene in this area, estimate their frequencies, and determine their possible deleterious effects.

## Materials and methods

### Design and setting of the study

This cross-sectional study was carried out at the Thalassemia Disease Centre at Jeen Hospital in Duhok City, Kurdistan Region, Iraq.

### Inclusion and exclusion criteria

The inclusion criteria for transfusion-dependent thalassemia (TDT) were: age at initial presentation <2 years, Hb levels 6–7 g/dl, and regular transfusion requirement [11]. Patients who did not fulfill the criteria for diagnosis of TDT, as set by the current study, were excluded.

### Statement of ethics

The Research Ethics Committee (Ministry of Health, Duhok Directorate General of Health, and Ministry of Higher Education, Duhok Polytechnic University) gave its approval to this study. On (31/07/2024), the permission letter reference number (31072024-6-26) was given. All patients or their guardians gave their written informed consent for the clinical specimens utilized in this investigation to be collected.

### Sample collection

The ethical board and scientific committee of Duhok Polytechnic University approved this study. This project involved 60 blood transfusion-dependent β-thalassemia (TDT) patients who registered at the Thalassemia Disease Center, Directorate General of Health, Duhok city, Kurdistan Region-Iraq, and sought treatment and regular follow-up at Jeen Hospital in Duhok from August 2024 to February 2025. All enrolled patients were interviewed; demographic data, ethnicity, native language, consanguinity among parents, age, gender, and disease history were obtained. The medical records of the patients were obtained from the medical record information system and reviewed. The age at diagnosis, age at first transfusion, and frequency of transfusions were scrutinized. Informed consent was obtained from all patients or their guardians. Blood samples were accessed and collected with EDTA anticoagulants from 5^th^ August 2024–27^th^ February 2025, for downstream laboratory examinations, DNA extraction, and genetic analysis [11,12].

### DNA extraction

The DNA was extracted from the blood using the Presto™ Mini gDNA Extraction Kit (Geneaid/Taiwan) following the manufacturer’s instructions. NanoDrope2000 spectrophotometer (Applied Biosystem) was used to assess the extracted DNA’s concentration and purity.

### PCR amplification of the *HBB* gene

All DNA samples underwent *in vitro* amplification by polymerase chain reaction (PCR) to amplify the entire beta-globin gene, encompassing three exons and two introns, using two sets of primers (Table 1) as described previously [13]. The typical 20μl PCR reaction mixture was formulated, comprising 1X TaqMaster mix (Promega), 10 pmol of forward primer (1μl), 10 pmol of reverse primer (1μl), and 20 ng of template DNA. The usual PCR settings for set I primers comprised an initial denaturation at 95°C for 3 minutes, followed by 30 cycles of 30 seconds at 95°C, 30 seconds at 58°C (annealing temperature), and 30 seconds at 72°C (extension temperature), concluding with a final extension of 3 minutes at 72°C. The thermal cycling of set II primers was conducted under conditions analogous to those of set I primers. The amplified products were stained using safe dye staining following electrophoresis on a 1.5% agarose gel in 1X TAE buffer, and the gel was electrophoresed at 75 volts for 45 minutes for verification and confirmation. The bands were visualized on a UV-transilluminator and captured using a photographic recording method (GBOX) [3].

**Table 1:** The set of primers used to amplify and sequence the beta-globin gene.

Gene	Primer Name	Sequence 5’-3’	Position*	Amplicon size
**B-Globin gene (*HBB*)**	HBB1-F	AGAAGAGCCAAGGACAGGTACG	61991−61990	760 bp
	HBB1-R	TGCAATCATTCGTCTGTTTCCC	62730-62751	
	HBB2-F	TCCCTAATCTCTTTCTTTCAGG	63190-63211	660 bp
	HBB2-R	TTTTCCCAAGGTTTGAACTAGC	63829-63849	

*Positions based on GenBank accession number U01317.

### Direct sequencing of the *HBB* gene

Following the guidelines provided by the manufacturer, the PCR products of the *HBB* gene were purified using a MinElute PCR Purification Kit (50) (Qiagen). Macrogen Inc. (Seoul, South Korea) employed an automated ABI3730XL genetic analyzer for subsequent Sanger sequencing of the purified PCR products. The primer pair of HBB1 and HBB2 was used separately in individual reactions for the bidirectional sequencing of the whole *HBB* gene using each of the forward and reverse primers, independently.

### Data analyses

Sequencing data were submitted to the GenBank using the BLAST method in the National Center for Biotechnology Information (NCBI) database to confirm the identity of the patients’ sequences. The patients’ sequences were compared with the NCBI Reference sequence (NG_000007.3, Homo sapiens chromosome 11, GRCh38.p13 Primary Assembly) using the Multalin alignment tool. Mutation frequency was determined by direct counting. Ithanet, HbVar, dbSNP IDs, and ClinVar databases were used for the identification and description of the mutations that had been identified in other populations [14]. A variant was considered novel if it was absent in all these major variant databases and population studies and had not been described in peer-reviewed literature. To calculate the probability of deleterious effects of novel mutation, different bioinformatics tools available online were used, viz EXPASY Translate tool (https://web.expasy.org/translate/) to translate the DNA sequence to protein sequence. The ACMG/AMP 2015 criteria were used for the pathogenicity classification of novel variants as pathogenic/likely pathogenic following the PVS1 (null variant in a gene where loss of function is a known disease mechanism) and PM2 (absence from population databases) criteria [15].

## Results

In the current study, a complete spectrum of β-globin gene (*HBB*) mutations was detected and reported. Of the 60 enrolled patients, 40 yielded high-quality, analyzable DNA sequence chromatograms. Out of them, 26 distinct mutations were identified, comprising 16 intronic and 10 exonic variants. Remarkably, seven of these variants were novel, reported here for the first time worldwide, and had never been reported in any of the pertinent databases, including dbSNP IDs, HGVS nomenclature, IthaGenes, HbVar, and ClinVar. Each novel mutation exhibits unique clinical significance, molecular effects, and different frequencies within the patient cohort (Table 2). The frequency, chromosomal location, and clinical relevance of each variation were determined using the current variant interpretation and classification guidelines (ACMG/AMP) and the aforementioned databases.

**Table 2:** *HBB* Gene Variants Found in the Study Cohort (n = 40) distributed across intronic and exonic regions.

Common Name	Transcript (NM_000518.5) and HGVS name	dbSNP Identifier	Genomic Locus (NG_000007.3)	Variant Type	Frequency (%)	ACMG/AMP Classification
IVS I-1 G > A	HBB:c.92 + 1G > A	rs33971440	g.70687G > A	Splice donor substitution	6/40 (15%)	Pathogenic
IVS I-5 (G > C)	HBB:c.92 + 5G > C	rs33915217	g.70691G > C	Splice region substitution	7/40 (17.5%)	Pathogenic
IVS I-5 (G > T)	HBB:c.92 + 5G > T	rs33915217	g.70691G > T	Splice region substitution	1/40 (2.5%)	Pathogenic
IVS I-6 (T > C)	HBB:c.92 + 6T > C	rs35724775	g.70692T > C	Splice region substitution	4/40 (10%)	Pathogenic
IVS I-6 (T > G)	HBB:c.92 + 6T > G	rs35724775	g.70692T > G	Splice region substitution	1/40 (2.5%)	Pathogenic
IVS I-110 G > A	HBB:c.93-21G > A	rs35004220	g.70796G > A	Intronic substitution	2/40 (5%)	Pathogenic
IVS I-129 + C Ins	HBB:c.92 + 129_92 + 130insC	Novel	g.70815_70816insC	Deep intronic insertion variant	1/40 (2.5%)	Likely Benign
IVS II-1 G > A	HBB:c.315 + 1G > A	rs33945777	g.71040G > A	Splice donor substitution	4/40 (10%)	Pathogenic
IVS II-16 G > C	HBB:c.315 + 16G > C	rs10768683	g.71055G > C	Intronic substitution	32/40 (80%)	Benign
IVS II-72 G > A	HBB:c.315 + 72 G > A	Novel	g.71111G > A	Intronic substitution variant	1/40 (2.5%)	VUS/Uncertain
IVS II-74 T > G	HBB:c.315 + 74T > G	rs7480526	g.71113T > G	Intronic substitution variant	3/40 (7.5%)	Benign
IVS II-81 (C > T)	HBB:c.315 + 81C > T	rs7946748	g.71120C > T	Intronic substitution variant	4/40 (10%)	Benign
IVS II-100 G > A	HBB:c.315 + 100G > A	rs899975457	g.71139G > A	Intronic substitution variant	1/40 (2.5%)	Benign
IVS II-579 G > A	HBB:c.316−272 G > A	Novel	g.71792G > A	Intronic substitution variant	1/40 (2.5%)	Benign
IVS II-666 (C > T)	HBB:c.316-185C > T	rs1609812	g.71705C > T	Intronic substitution variant	31/40 (77.5%)	Benign
IVS II-763 + C Ins	HBB:c.316−87_ 316−88insC	Novel	g.71607_71608insC	Intronic insertion variant	2/40 (5%)	Likely pathogenic
Cd2 CAT > CAC	HBB:c.9 T > C	rs713040	g.70603T > C	silent substitution variant	25/40 (62.5%)	Benign
Cd5 -CT	HBB:c.17_18delCT	rs63750729	g.70611_70612del	Frameshift deletion	7/40 (17.5%)	Pathogenic
Cd6 GAG > GTG	HBB:c.20A > T	rs334	g.70614A > T	Missense variant (Glu > Val)	1/40 (2.5%)	Pathogenic (sickle cell disease)
Cd8 AAG > AGG	HBB:c.26A > G	rs33932981	g.70620A > G	Missense variant (Lys > Arg)	1/40 (2.5%)	Pathogenic
Cd39 CAG > TAG	HBB:c.118C > T	rs11549407	g.70842C > T	Nonsense variant (Gln>STOP)	2/40 (5%)	Pathogenic
Cd44 TCC > TTC	HBB:c.134C > T	--	g.70858C > T	Missense variant (Ser > Phe)	1/40 (2.5%)	Pathogenic
Cd44 TCC > TCT	HBB:c.135C > T	Novel	g.70859C > T	silent substitution variant (Ser > Ser)	1/40 (2.5%)	Benign
Cd44 -C	HBB:c.135delC	rs80356820	g.70859del	Frameshift deletion	1/40 (2.5%)	Pathogenic
Cd47 -G	HBB:c.142delG	Novel	g.70866delG	Frameshift deletion	1/40 (2.5%)	Pathogenic
Cd118 -TT	HBB:c.355_356delTT	Novel	g.71929_71930delTT.	Frameshift deletion	1/40 (2.5%)	Pathogenic

Abbreviations: HGVS: Human Genome Variation Society, dbSNP: Database for Single Nucleotide Polymorphisms.

Note: All variants are reported using HGVS nomenclature with RefSeq transcript NM_000518.5 and RefSeqGene NG_000007.3 on GRCh38.p13. The ACMG/AMP 2015 classification is based on predicted pathogenicity. The known variations were evaluated using the existing variation field (dbSNP rsIDs) and corroborated with ClinVar and GnomeAD.

**Fig 1:**
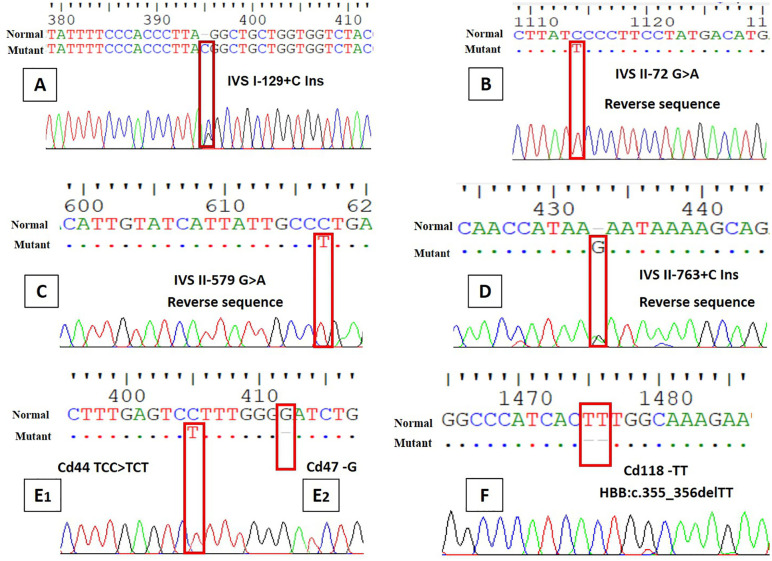
Chromatograms of the novel *HBB* gene mutations identified in this study.

Starting with the intronic variants, a total of 16 intronic mutations were found in the β-globin gene’s introns 1 (IVS-I) and 2 (IVS-II). Intron 1 contained seven variants that mostly affected the splice donor and splice region: IVS I-5 G > C (HBB:c.92 + 5G > C, rs33915217) was the most common, occurring in 17.5% of samples, followed by IVS I-1 G > A (HBB:c.92 + 1G > A, rs33971440) and IVS I-6 T > C (HBB:c.92 + 6T > C, rs35724775) observed in 15% and 10% of cases, respectively. These substitutions are known to disrupt normal splicing and are considered pathogenic. One case (2.5%) contained a novel insertion, IVS I-129 + C Ins (HBB:c.92 + 129_92 + 130insC), which was deemed likely benign because it was located in the deep region of intron 1 (Fig 1A).

Additionally, Intron 2 was found to have nine variations. Of which, IVS II-16 G > C (rs10768683) was the most prevalent variant overall, occurring in 80% of patients, yet being regarded as benign. IVS II-666 (C > T) (rs1609812) was also identified in 77.5% of instances and is categorized as benign. Though less common, other variations, including IVS II-1 G > A (10%) and IVS II-763 + C Ins (5%), which have the potential to be pathogenic, were also present. The discovery of three new intronic variants, including IVS II-72 G > A (HBB:c.315 + 72 G > A) (Fig 1B), IVS II-579 G > A (HBB:c.316−272 G > A) (Fig 1C), and IVS II-763 + C Ins (HBB:c.316−87_ 316−88insC) (Fig 1D), suggests even more variation in non-coding areas.

In the gene’s coding regions (exons 1–3), ten genetic variations were found. These included a variety of mutation types, such as frameshift, missense, nonsense, and silent (synonymous) mutations. The prevalence of these variations in the research samples and their anticipated effects on protein function differ. There were four different variations found in exon 1. A silent alteration at Cd2 CAT > CAC (HBB:c.9T > C) was the most common, occurring in 62.5% of samples and deemed benign. Missense mutations like Cd6 GAG > GTG (HBB:c.20A > T, p.Glu6Val), the classical cause of sickle cell disease (HbSS) mutation, and frameshift deletions like Cd5-CT (HBB:c.17_18delCT) were found at lower frequency (2.5–17.5%) and were deemed pathogenic because of their disruptive effects on protein structure and function.

Within exon 2, five different variations were found. Notably, 5% of the samples had the Cd39 CAG > TAG (HBB:c.118C > T), a nonsense mutation that introduced a premature stop codon to the β-globin chain. Remarkably, codon 44 showed a range of disease-causing mutations among the studied cohort, including a frameshift deletion (HBB:c.135delC) and missense alterations (HBB:c.134C > T) in addition to a novel synonymous (silent) variation (Cd44 TCC > TCT; HBB:c.135C > T). Additionally, another novel frameshift deletion (Cd47 –G; HBB:c.142delG) was detected in 2.5% of the samples, which was deemed as pathogenic.

Furthermore, a new frameshift deletion mutation in exon 3, called Cd118-TT (HBB:c.355_356delTT), which disrupts the reading frame, was identified in 2.5% of patients. This mutation likely results in a shortened and inoperable β-globin protein. It is classified as pathogenic because of its expected impact on the structure and function of the β-globin protein.

The identification and reporting of seven unique mutations, out of the 26 discovered variants, that had not been previously reported in public databases like dbSNP, ClinVar, HbVar, and other pertinent databases, is remarkable. The *HBB* gene has three new intronic variations, all of which were found in non-coding areas that may have an impact on gene regulation, RNA splicing, or transcript stability. A single-nucleotide cytosine insertion in intron 1 called IVS I-129 + C Ins (HBB:c.92 + 129_92 + 130insC) is located close to regulatory elements and has the potential to interfere with splicing enhancer or silencer sites. Intron 2 has the guanine-to-adenine alteration known as IVS II-72 G > A (HBB:c.315 + 72G > A), which is situated 72 nucleotides downstream of exon 2. This area could be important for identifying intron-exon boundaries. Intron 2 has the guanine-to-adenine transition IVS II-579 G > A (HBB:c.316-272G > A), which is located 272 nucleotides upstream of exon 3 and may have an impact on intronic regulatory motifs. Specifically, IVS II-763 + C Ins and IVS I-129 + C Ins were deemed to be probable pathogenic and require further research. Furthermore, four new variations were found in the exonic areas. A silent mutation at codon 44, Cd44 TCC > TCT (HBB:c.135C > T) (Fig 1E_1_), may affect codon use or mRNA stability but does not change the amino acid (Ser > Ser). The β-globin chain is expected to be shortened and rendered inoperable by the frameshift deletion at codon 47 known as Cd47 -G (HBB:c.142delG) (Fig 1E_2_). The pathogenic mutation of Cd118 -TT (HBB:c.355_356delTT) (Fig 1F) was another frameshift loss in exon 3. The disruptive character of this two-nucleotide loss frameshift deletion and its anticipated effect on protein synthesis led to its classification as pathogenic.

In this study, a total of 23 different genotypes were identified among the 40 β-thalassemia patients analyzed, which indicated a diverse mutation spectrum. The genotypic pattern showed that 92.5% (37/40) of the investigated β-thalassemia subjects were compound heterozygous for two or more different mutations (S1 Table), suggesting that complex compound-heterozygous genotypes are highly prevalent. Within this 40-patient cohort, the *HBB* genotype burden varied from 1 to 5 different variations per patient (mean ± SD = 3.60 ± 1.20; median = 4). Of these, 10% (4/40) of patients carried two different mutations, 25% (10/40) had three, and 27.5% (11/40) had five distinct variants. Additionally, the most frequent group, 30% (12/40), had four various mutations. In contrast, simple homozygosity was uncommon, in which only 3 patients (7.5%) were homozygous for a single pathogenic variant, of which two cases had IVS I-5 G > C and one case had Cd8 A > G genotype. These findings were consistent with transfusion-dependent β-thalassemia and demonstrate a high frequency of compound heterozygosity in the studied population. Furthermore, among the compound heterozygous profiles, the most prevalent transfusion-dependent genotype was (Cd2 T > C, Cd5 –CT, IVS II-16 G > C, IVS II-666 C > T) observed in 15% of the studied cohort, accompanied by (Cd2 T > C, IVS II-16 G > C, IVS II-666 C > T) in 10%, and each of (Cd2 T > C, IVS I-110 G > A, IVS II-16 G > C, IVS II-74 T > G, IVS II-666 C > T) and (Cd2 T > C, IVS I-1 G > A, IVS II-16 G > C, IVS II-666 C > T) in 7.5%, respectively, indicating recurrent combinations. Other less frequent genotypic combinations (5% frequency) were (Cd2 T > C, IVS I-1 G > A, IVS II-1 G > A, IVS II-16 G > C, IVS II-666 C > T), (IVS I-6 T > C, IVS II-16 G > C, IVS II-666 C > T), and (IVS II-16 G > C, IVS II-666 C > T). However, 40% of the identified genotypes, which ranged from a single homozygous variant to five different variants in compound heterozygosity, were unique to single patients. Additionally, 17.5% of patients (7/40) carried at least one novel variant in the dataset. Altogether, these findings showed a significant mutation burden per patient with notable allelic heterogeneity.

## Discussions

The current study broadens the genetic epidemiology of β-thalassemia in Iraq by identifying 26 different HBB variations in a patient cohort under study, including seven previously unknown alterations. The global pattern of a relatively small core of highly prevalent mutations accompanied by a long “tail” of population- or family-specific changes is reflected in this breadth of allelic diversity. According to a recent synthesis, over 350 β-thalassemia alleles have been described worldwide, but only about 20 of these account for more than 80% of cases [16]. Therefore, our findings support the idea of significant regional heterogeneity and highlight the need for region-specific mutation panels for prenatal diagnosis and carrier identification.

In our dataset, three classical splice-site mutations (IVS I-5 G > C, IVS I-1 G > A, and IVS I-6 T > C) together accounted for 42.5% of the pathogenic alleles. These lesions have been reported to be similarly prevalent in the Iraqi premarital screening program (IVS I-5 G > C = 12.4%; IVS I-6 T > C = 8.3%), as well as in a previous Baghdad study when IVS I-5 G > C came in third rank overall [17]. On a regional level, IVS I-5 G > C is also the most prevalent allele in Bangladesh (81%) [18], Pakistan (44%) [19], and portions of Southeast Asia and Thailand (34%–40%) [4,20], indicating a South-to-West trend that most likely reflects historical gene flow along trade routes. Transfusion-dependent symptoms in our cohort are explained by these changes, which result in β⁰- or severe β ⁺ -thalassemia transcripts by eliminating or weakening canonical splice donor sites.

Conversely, the most common alleles overall were IVS II-16 G > C (80%) and IVS II-666 C > T (77.5%), both of which are regarded as benign polymorphisms. Similar elevated carriage rates (60 percent for IVS II-16 G > C) have been seen in healthy Bangladeshi controls [18]. However, there is now emerging evidence that these variations can regulate illness when co-inherited with severe alleles or Hb S. Case reports demonstrate the change of basic sickle-cell trait to Hb S/β-thalassemia intermedia when either polymorphism is present [21,22]. While the transfusion reliance of participants is mostly explained by the presence of severe β⁰ or splice-abolishing alleles, the occurrence of supposed benign polymorphisms (e.g., IVS II-16 G > C) may operate as disease modifiers by changing the α/β-globin imbalance or residual β-chain production. Deep-intron variations in other populations have been suggested to have similar modifying functions [22]. Although the current study may not have been powered to analyze such epistatic interactions, the extremely high background frequency necessitates caution when interpreting compound genotypes in clinical settings.

We found three intronic alterations that had not been reported before: IVS II-129 insC, IVS II-72 G > A, and IVS II-579 G > A. All of them are located in deep-intron areas that are enriched for cryptic splice enhancers and silencers. This suggests that they may disrupt or generate binding sites for hnRNP A1 or SR-protein, and as such, they should be studied using functional minigene splicing tests. The ACMG/AMP guidelines [15,23] support the preliminary “likely pathogenic” diagnosis of IVS II-763 insC, a fourth new variation that was found in 5% of patients and consistently co-segregated with severe anemia.

In the current investigation, ten exonic mutations have been reported in the *HBB* gene. Collectively, they ranged from subtle variations that have little to no effect on phenotype to severe mutations that cause β-thalassemia or sickle cell disease. With worldwide allele frequencies as high as 0.8%, the silent c.9 T > C (codon 2) variant reached 62.5%; ClinVar and gnomAD classify this variant as benign [15,24]. Its significant local incidence highlights the need to avoid incorrectly categorizing synonymous alterations as disease-causing in diagnostic reports and implies linkage disequilibrium with a Middle-Eastern *HBB* haplotype [25].

The study revealed that 17.5% of the participants had the famous HBB:c.20A > T [Glu6Val (Hb S)] mutation, highlighting the persistent gene flow between the northern Iraqi β-thalassemia and sickle-cell endemic areas. Intermarriages, migrations, and demographic exchanges between tribes living in these overlapping genetic belts are all reflected in this discovery. For the therapy of hemoglobinopathies in the area, the comparatively high frequency of Hb S points to a considerable mixing that might have both clinical and epidemiological consequences. With almost 5% of the cases, the codon 39 (CAG > TAG) nonsense mutation was found to be the second most common coding lesion. Its well-established position as the second most prevalent β-thalassemia allele in the larger Mediterranean basin is in line with this. Interestingly, in Sardinia and several regions of North Africa, this mutation is also the most common form that causes β-thalassemia [26,27]. Consistent with earlier research carried out in Baghdad [17], the current cohort once more exhibited the characteristic frameshift mutations Cd5 delCT and Cd44 delC. The continuous reporting of these recurrent mutations in previous regional studies suggests that they may be enriched in the local population. Three new mutations were found in addition to these known variations: a silent substitution at Cd44 (c.135C > T) and frameshifts Cd47 delG and Cd118 delTT. Premature termination codons are expected to be introduced by all these pathogenic mutations, most likely leading to nonsense-mediated mRNA decay (NMD). A β⁰-thalassemia phenotype, which is defined by the total lack of functional β-globin production, is effectively caused by this degradation process, which stops the synthesis of shortened β-globin chains. This discovery highlights the significance of thorough molecular screening in genetic diagnosis and counselling and adds to the growing mutational spectrum of β-thalassemia in the Iraqi population, especially the new variations.

Through full HBB locus sequencing, we identified 26 unique variations, including seven entirely new alterations in a current cohort from Duhok, Kurdistan Region, Iraq. The regional allele inventory now has over 40 mutations, which is wider than any single-center Kurdish research to date. Just 11–22 mutations were usually detected in earlier studies that relied on strip hybridization or restricted Sanger panels [9]. Thus, our findings highlight the need to update routine diagnostic panels and the discovery capability of full-gene methods.

The IVS-II-1 G > A and IVS-I-6 T > C continue to be the dominant pathogenic alleles across all Kurdish provinces. They accounted for 25% of the mutations in the present cohort and for 24% to 43% of the mutations in the successive Duhok studies from 2006 to 2021 [9,12], 39% of the transfusion-dependent patients from Dohuk/Erbil [28], and 54% of the β-thalassemia intermedia alleles [29]. Notwithstanding methodological and phenotypic variations, the 2020 Sulaymaniyah audit verified the same hierarchy (IVS-II-1 = 35.7%, IVS-I-6 = 18%) [11]. Strong founder effects that are strengthened by consanguinity are supported by this temporal stability. However, this iconic image has been altered by two new datasets. In comparison to 2006 data, the 2021 “updated spectrum” research revealed that the influx of Yazidi and other displaced families roughly increased the prevalence of IVS-I-110 G > A and codon8 -AA [12]. Additionally, we found a little increase in these alleles (4–6%), which points to ongoing gene flow in the Mosul–Duhok corridor. And, after comprehensive sequencing took the place of strip testing, a 2023 Sanger-sequencing survey found that 59% of patients had IVS-II-666 C > T and IVS-II-16 G > C, along with five new deep-intron alterations [3]. This result is consistent with our own dataset (77–80%), demonstrating how previously “invisible” polymorphisms become apparent when the full gene is examined. Furthermore, the β-thalassemia-intermedia cohort, where the moderate IVS-I-6 T > C allele alone explained one-third of cases [29], contrasts with transfusion-dependent cohorts, which are more likely to have severe β⁰ or splice-abolishing alleles, for example, 88% of variants explained by seven mutations in the 2010 series [28].

Our study adds seven new variations to the Kurdish mutation pool that had not been reported before. Notably, we uncovered a codon 118 delTT frameshift that independently supports a 2021 solitary case account [10], albeit with a different position at the same codon (355_356 vs. 356_357). Both result in a frameshift at codon 118 and most likely produce the same shortened/nonfunctional β-globin chain. This confirms that this β⁰ allele is a low-frequency founder mutation rather than an anecdote. Five further unique intronic insertions and deletions were included in the Sulaymaniyah 2023 investigation [3], highlighting the fact that non-coding areas are still understudied sources of pathogenic alteration. Although it still targets the 23 “common” mutations, the Iraqi premarital kit, which has been frozen since 2010, would miss 29% of the alleles in our cohort and 41% of the most recent Sulaymaniyah series. First-tier sensitivity would increase to >90% with the addition of IVS-I-110 G > A, codon8 -AA, high-frequency intronic SNPs, and the seven new pathogenic mutations reported here. Reflex next-generation sequencing, which has already been implemented in a few of the UK screening hubs, would future-proof detection in the face of ongoing migration and mutational drift when resources allow.

The high frequency of compound heterozygosity in β-thalassemia major was consistent with transfusion-dependent β-thalassemia in the studied population. Additionally, the findings pointed to compound heterozygosity as the primary cause of the observed genetic architecture and phenotypic heterogeneity, including transfusion dependency and severity levels. The high level of compound heterozygosity reported in the current population aligns with previous studies [30,31], which also reported a similar predominance of compound heterozygous genotypes in other β-thalassemia cohorts. This may suggest a multifactorial inheritance pattern, in which several mutations contribute to the disease manifestation. Additionally, the recurrent genotypic combinations reported in this study suggest shared ancestry, or the population may have experienced founder effects. The presence of these recurrent mutations in different groups suggests that they may contribute to the pathophysiology of β-thalassemia [32]. Furthermore, the study population’s genetic diversity was demonstrated by the 37.5% of patients who had unique genotypes found. Other investigations have reported similar findings, with a sizable percentage of patients displaying distinct genotypic profiles [33]. Overall, the genotypic clustering and zygosity pattern findings of this study demonstrated the importance of precise genotyping for genetic counseling, diagnosis, and prognosis.

## Conclusions

Altogether, by identifying a diverse range of *HBB* variations, including 17 (65%) variants categorized as pathogenic or potentially pathogenic and several benign or unique polymorphisms, such as deep-intron and frameshift events, this work expands the Iraqi β-thalassemia mutation database. The detection of a remarkably diverse mutation spectrum and high prevalence of compound heterozygosity among the β-thalassemia patients is indicative of intricate allelic interactions that lead to the severe, transfusion-dependent clinical phenotypes observed. The development of locus-specific therapies tailored to the allelic landscape of Iraq should be guided by these insights, which should be incorporated into regional diagnostic algorithms to enhance carrier detection and reproductive risk assessment. To establish the identified new mutations’ clinical significance and ascertain their specific biological impact, further functional validation and segregation analysis are recommended.

## Supporting information

S1 Table Supporting information. (DOCX)

## References

[1] Huang P, Peslak SA, Ren R, Khandros E, Qin K, Keller CA, et al. HIC2 controls developmental hemoglobin switching by repressing BCL11A transcription. Nat Genet. 2022;54(9):1417–26. doi: 10.1038/s41588-022-01152-6 35941187 PMC9940634

[2] Mann S, Raikina E, Yunusova I. The spectrum of genetic variants of the α- and β-globin clusters in patients with hemoglobinopathies living in the Republic of Dagestan. Pediatr Hematol Oncol Immunopathol. 2020;19(3):50–3.

[3] Ahmad N, Karim H, Rasool L, Ali K, Mahmood M, Osman T, et al. A spectrum of β-thalassemia mutations in sulaimani province of iraq: identification of novel mutations. JSMC. 2023;13(4):7. doi: 10.17656/jsmc.10435

[4] Boonyawat B, Monsereenusorn C, Traivaree C. Molecular analysis of beta-globin gene mutations among Thai beta-thalassemia children: results from a single center study. Appl Clin Genet. 2014;7:253–8. doi: 10.2147/TACG.S73058 25525381 PMC4266330

[5] Ulasli M, Oztuzcu S, Kirkbes S, Bay A, Igci YZ, Bayraktar R, et al. Novel Βeta (β)-Thalassemia Mutation in Turkish Children. Indian J Hematol Blood Transfus. 2015;31(2):218–22. doi: 10.1007/s12288-014-0380-6 25825561 PMC4375154

[6] Xu L, Mao A, Liu H, Gui B, Choy KW, Huang H, et al. Long-Molecule Sequencing: A New Approach for Identification of Clinically Significant DNA Variants in α-Thalassemia and β-Thalassemia Carriers. J Mol Diagn. 2020;22(8):1087–95. doi: 10.1016/j.jmoldx.2020.05.004 32473995

[7] Eissa AA, Kashmoola MA, Atroshi SD, Al-Allawi NAS. Molecular Characterization of β-Thalassemia in Nineveh Province Illustrates the Relative Heterogeneity of Mutation Distributions in Northern Iraq. Indian J Hematol Blood Transfus. 2015;31(2):213–7. doi: 10.1007/s12288-014-0369-1 25825560 PMC4375141

[8] Hamid M, Zargan Nezhad E, Keikhaei B, Galehdari H, Saberi A, Sedaghat A, et al. Two Novel and Five Rare Mutations in the Non Coding Regions of the β-Globin Gene in the Iranian Population. Hemoglobin. 2020;44(4):225–30. doi: 10.1080/03630269.2020.1790384 32672086

[9] Al-Allawi NAS, Jubrael JMS, Hughson M. Molecular characterization of beta-thalassemia in the Dohuk region of Iraq. Hemoglobin. 2006;30(4):479–86. doi: 10.1080/03630260600868097 16987803

[10] Atroshi SD, Al-Allawi N, Chui DHK, Najmabadi H, Khailany RA. A Novel β0-Thalassemia Mutation, HBB: c.356_357delTT [Codon 118 (-TT)] in an Iraqi Kurd. Hemoglobin. 2021;45(3):212–4. doi: 10.1080/03630269.2021.1941082 34167424

[11] Amin S, Jalal S, Ali K, Rasool L, Osman T, Ali O, et al. Molecular Characterization and Disease-Related Morbidities of β-Thalassemia Patients from the Northeastern Part of Iraq. Int J Gen Med. 2020;13:1453–67. doi: 10.2147/IJGM.S277947 33335418 PMC7737013

[12] Atroshi SD, Al-Allawi NAS, Eissa AA. Updated Molecular Spectrum of β-Thalassemia Mutations in Duhok Province, Northern Iraq: Ethnic Variation and the Impact of Immigration. Hemoglobin. 2021;45(4):239–44. doi: 10.1080/03630269.2021.1984250 34794358

[13] Mirasena S, Shimbhu D, Sanguansermsri M, Sanguansermsri T. The spectrum of β-thalassemia mutations in Phitsanulok Province: development of multiplex ARMS for mutation detection. Asian Health Sci Technol Rep. 2007;15(1):43–53.

[14] Chauhan RP, Gordon ML. Review of genome sequencing technologies in molecular characterization of influenza A viruses in swine. J Vet Diagn Invest. 2022;34(2):177–89. doi: 10.1177/10406387211068023 35037523 PMC8921814

[15] Richards S, Aziz N, Bale S, Bick D, Das S, Gastier-Foster J, et al. Standards and guidelines for the interpretation of sequence variants: a joint consensus recommendation of the American College of Medical Genetics and Genomics and the Association for Molecular Pathology. Genet Med. 2015;17(5):405–24. doi: 10.1038/gim.2015.30 25741868 PMC4544753

[16] Rao E, Kumar Chandraker S, Misha Singh M, Kumar R. Global distribution of β-thalassemia mutations: An update. Gene. 2024;896:148022. doi: 10.1016/j.gene.2023.148022 38007159

[17] Al-Allawi NAS, Al-Mousawi BMS, Badi AIA, Jalal SD. The spectrum of β-thalassemia mutations in Baghdad, Central Iraq. Hemoglobin. 2013;37(5):444–53. doi: 10.3109/03630269.2013.810641 23826747

[18] Sultana GNN, Begum R, Akhter H, Shamim Z, Rahim MA, Chubey G. The complete spectrum of β-thalassemia mutations in Bangladeshi population. Austin Biomark Diagn. 2016;3(1):1024.

[19] Usman M, Moinuddin M, Ghani R, Usman S. Screening of Five Common Beta Thalassemia Mutations in the Pakistani Population: A basis for prenatal diagnosis. Sultan Qaboos Univ Med J. 2009;9(3):305–10. doi: 10.18295/2075-0528.2805 21509314 PMC3074786

[20] Nuinoon M, Rattanaporn P, Benjchareonwong T, Choowet A, Suwanno K, Saekoo N, et al. Genetic predictions of life expectancy in southern Thai patients with β0-thalassemia/Hb E. Biomed Rep. 2022;16(6):52. doi: 10.3892/br.2022.1535 35620315 PMC9112403

[21] Uçucu S, Karabıyık T, Azik FM. IVS-II-16 (G>C) (HBB: c.315 + 16G>C) or IVS-II-666 (C>T) (HBB: c.316-185C>T) Mutations Trigger an Hb S (HBB: c.20A>T)/β+-Thalassemia Phenotype in an Hb S Trait Patient. Hemoglobin. 2021;45(4):225–7. doi: 10.1080/03630269.2021.1965620 34396882

[22] Chauhan W, Fatma R, Zaka-Ur-Rab Z, Afzal M. Direct sequencing of β-globin gene reveals a rare combination of two exonic and two intronic variants in a β-thalassemia major patient: a case report. J Med Case Rep. 2022;16(1):362. doi: 10.1186/s13256-022-03605-2 36209112 PMC9548154

[23] Preston CG, Wright MW, Madhavrao R, Harrison SM, Goldstein JL, Luo X, et al. ClinGen Variant Curation Interface: a variant classification platform for the application of evidence criteria from ACMG/AMP guidelines. Genome Med. 2022;14(1):6. doi: 10.1186/s13073-021-01004-8 35039090 PMC8764818

[24] Leiden Open Variation Database (LOVD). Variants/604091#00000017 [database record]. Leiden Open Variation Database; n.d. [Accessed 2025 Jun 25]. Available from: https://databases.lovd.nl/shared/variants/604091#00000017

[25] Pines M, Sheth S. Clinical Classification, Screening, and Diagnosis in Beta-Thalassemia and Hemoglobin E/Beta-Thalassemia. Hematol Oncol Clin North Am. 2023;37(2):313–25. doi: 10.1016/j.hoc.2022.12.003 36907605

[26] Thein SL. The molecular basis of β-thalassemia. Cold Spring Harb Perspect Med. 2013;3(5):a011700. doi: 10.1101/cshperspect.a011700 23637309 PMC3633182

[27] Kamilia B, Mouloud Y, Moez G, Fadhila B, Ilhem BC, Wiem M, et al. Effects of codon 39 (C>T) mutation on hematological parameters in children with β-thalassemia major in Batna, Algeria. Curr Pediatr Res. 2016;20(1):203–8.

[28] Al-Allawi NAS, Hassan KMA, Sheikha AK, Nerweiy FF, Dawood RS, Jubrael J. β-Thalassemia Mutations among Transfusion-Dependent Thalassemia Major Patients in Northern Iraq. Mol Biol Int. 2010;2010:479282. doi: 10.4061/2010/479282 22110956 PMC3218307

[29] Al-Allawi NAS, Jalal SD, Mohammad AM, Omer SQ, Markous RSD. β -thalassemia intermedia in Northern Iraq: a single center experience. Biomed Res Int. 2014;2014:262853. doi: 10.1155/2014/262853 24719849 PMC3955643

[30] Chaudhary S, Dhawan D, Bagali PG, S.Chaudhary P, Chaudhary A, Singh S, et al. Compound heterozygous β+ β0 mutation of HBB gene leading to β-thalassemia major in a Gujarati family — A case study. Molecular Genetics and Metabolism Reports. 2016;7:51–3. doi: 10.1016/j.ymgmr.2016.04.002 27134826 PMC4834677

[31] Wu H-P, Lin C-L, Chang Y-C, Wu K-H, Lei R-L, Peng C-T, et al. Survival and complication rates in patients with thalassemia major in Taiwan. Pediatr Blood Cancer. 2017;64(1):135–8. doi: 10.1002/pbc.26181 27571924

[32] Khan AM, Al-Sulaiti AM, Younes S, Yassin M, Zayed H. The spectrum of beta-thalassemia mutations in the 22 Arab countries: a systematic review. Expert Rev Hematol. 2021;14(1):109–22. doi: 10.1080/17474086.2021.1860003 33317346

[33] Huang T-L, Zhang T-Y, Song C-Y, Lin Y-B, Sang B-H, Lei Q-L, et al. Gene Mutation Spectrum of Thalassemia Among Children in Yunnan Province. Front Pediatr. 2020;8:159. doi: 10.3389/fped.2020.00159 32351918 PMC7174584

