# Supplementary material for: Complete molecular spectrum of β-globin gene mutations via direct sequencing identifies seven novel variants in β-thalassemia major
Source: PLoS One. 2025 Nov 7;20(11):e0336610. doi: 10.1371/journal.pone.0336610 (PMC12594352; doi:10.1371/journal.pone.0336610)
Supplement: S1 Table — (DOCX) [file pone.0336610.s001.docx]

**Supporting information**

**Table S1:** Genotypic Spectrum, frequencies, and compound heterozygosity in 40 HBB gene patients.

| Genotype pattern (HBB variants) | No. of patients (n) | Frequency (%) | No. of mutations | Zygosity / Notes |
| --- | --- | --- | --- | --- |
| Cd2 T>C, Cd5 –CT, IVS II-16 G>C, IVS II-666 C>T | 6 | 15.0 | 4 | Compound heterozygote |
| Cd2 T>C, IVS II-16 G>C, IVS II-666 C>T | 4 | 10.0 | 3 | Compound heterozygote |
| Cd2 T>C, IVS I-110 G>A, IVS II-16 G>C, IVS II-74 T>G, IVS II-666 C>T | 3 | 7.5 | 5 | Compound heterozygote |
| Cd2 T>C, IVS I-1 G>A, IVS II-16 G>C, IVS II-666 C>T | 3 | 7.5 | 4 | Compound heterozygote |
| Cd2 T>C, IVS I-1 G>A, IVS II-1 G>A, IVS II-16 G>C, IVS II-666 C>T | 2 | 5.0 | 5 | Compound heterozygote |
| IVS I-6 T>C, IVS II-16 G>C, IVS II-666 C>T | 2 | 5.0 | 3 | Compound heterozygote |
| IVS I-5 G>C | 2 | 5.0 | 1 | Simple homozygote |
| IVS II-16 G>C, IVS II-666 C>T | 2 | 5.0 | 2 | Compound heterozygote |
| IVS I-5 G>C, IVS II-16 G>C, IVS II-666 C>T | 1 | 2.5 | 3 | Compound heterozygote |
| IVS I-6 T>C, Cd39 C>T, IVS II-81 C>T, IVS II-100 G>A | 1 | 2.5 | 4 | Compound heterozygote |
| IVS II-16 G>C, IVS II-72 G>A (Novel) , IVS II-666 C>T | 1 | 2.5 | 3 | Compound heterozygote; novel variant |
| IVS I-6 T>G, IVS II-81 C>T, Cd118 -TT (Novel) | 1 | 2.5 | 3 | Compound heterozygote; novel variant |
| Cd2 T>C, Cd44 C>T, IVS II-16 G>C, IVS II-666 C>T | 1 | 2.5 | 4 | Compound heterozygote |
| IVS I-5 G>C, Cd44 C>T (Novel), Cd47 –G (Novel), IVS II-16 G>C, IVS II-666 C>T | 1 | 2.5 | 5 | Compound heterozygote; two novel variants |
| Cd2 T>C, Cd44 –C, IVS II-16 G>C, IVS II-74 T>G, IVS II-666 C>T | 1 | 2.5 | 5 | Compound heterozygote |
| Cd2 T>C, IVS I-1 G>A, IVS I-5 G>T, IVS II-16 G>C, IVS II-666 C>T | 1 | 2.5 | 5 | Compound heterozygote |
| Cd2 T>C, IVS II-1 G>A, IVS II-16 G>C, IVS II-579 G>A (Novel), IVS II-666 C>T | 1 | 2.5 | 5 | Compound heterozygote; novel variant |
| Cd2 T>C, Cd6 A>T, IVS II-1 G>A, IVS II-16 G>C, IVS II-666 C>T | 1 | 2.5 | 5 | Compound heterozygote |
| Cd2 T>C, IVS I-5 G>C, IVS II-16 G>C, IVS II-666 C>T | 1 | 2.5 | 4 | Compound heterozygote |
| Cd8 A>G | 1 | 2.5 | 1 | Simple homozygote |
| IVS I-6 T>C, IVS I-129+C Ins (Novel), IVS II-81 C>T | 1 | 2.5 | 3 | Compound heterozygote; novel variant |
| Cd39 C>T, IVS II-81 C>T | 1 | 2.5 | 2 | Compound heterozygote |
| Cd2 T>C, Cd5 –CT, IVS II-16 G>C, IVS II-666 C>T, IVS II-763+C Ins (Novel) | 1 | 2.5 | 5 | Compound heterozygote; novel variant |
| IVS I-5 G>C, IVS II-763+C Ins (Novel) | 1 | 2.5 | 2 | Compound heterozygote; novel variant |
