# Supplementary material for: Complete molecular spectrum of β-globin gene mutations via direct sequencing identifies seven novel variants in β-thalassemia major
Source: PLoS One. 2025 Nov 7;20(11):e0336610. doi: 10.1371/journal.pone.0336610 (PMC12594352; doi:10.1371/journal.pone.0336610)
Supplement: Attachment — Submitted filename: Response to Reviewers.docx [file pone.0336610.s003.docx]

**Response to Reviewers**

We sincerely thank the reviewer for their meticulous evaluation and insightful comments, which have significantly strengthened the clarity, coherence, and scientific rigor of the manuscript. All revisions made in response to the reviewers’ suggestions are clearly highlighted in yellow in the Revised Manuscript with Track Changes.

**Journal requirements:**

Thank you for your guidance and for the helpful reminder concerning the journal’s requirements. We truly appreciate the editorial board’s efforts and support.

**Comment 1:** PLOS ONE's style requirements, including those for file naming and the style templates.

**Answer:** We revised the manuscript and ensured that it fully complied with PLOS ONE's style requirements, including the file naming conventions and following the PLOS ONE style templates guidelines. Additionally, all references are now fully compliant with the Vancouver (ICMJE) format used in biomedical journals.

**Comment 2:** Data Availability Statement.

**Answer:** We have confirmed that our submission contains all raw data required to replicate the results of our study, and the Data Availability Statement accurately reflects this.

**Comment 3:** Data sharing plan.

**Answer:** We reviewed our data sharing plan and ensured that all required data are available in accordance with PLOS ONE’s open data policy. Our Data Availability Statement has been updated accordingly. We appreciate your guidance on this matter.

**Comment 4:** Recommendation to cite specific previously published works.

**Answer:** We carefully reviewed the ACMG/AMP guidelines recommended by the reviewers and evaluated their relevance to our study. The appropriate works have been cited in the methodology section, and we are grateful for the constructive suggestions provided.

**Response to Reviewers' Comments:**

**Reviewer#1:**

Thank you very much for your insightful and constructive comments. We appreciate your suggestion regarding the pathogenicity classification system. In response, we have now clearly indicated that the ACMG (2015) guideline was used for variant interpretation, as it represents the current standard in the field. We have also revised the manuscript to replace non-standard terms such as “benign mutations” with the appropriate terminology recommended by ACMG/AMP. Furthermore, we have updated the reporting of novel variants to follow standard nomenclature, including NM numbers, nucleotide changes, and chromosomal position to ensure clarity and facilitate database verification.

**Reviewer#2:**

We sincerely thank the Reviewer for his/her thoughtful and encouraging comments on our manuscript. We greatly appreciate the recognition of the clinical relevance and contribution of our study to the understanding of β-thalassemia in the Kurdish population of Iraq.

1. **Methods:** Inclusion of the ACMG/AMP guidelines.

In response to your suggestion, we have now specified the exact criteria (e.g., PVS1, PM2, PP3, BS1) applied to classify the novel variants as pathogenic or likely pathogenic. This information has been added to the Methods section for greater transparency and clarity.

2. **Results:**

We appreciate your valuable suggestion to present the genotype distribution and its clinical correlation. Accordingly, we have revised the Results section to include a new summary detailing the genotypes of the patients, including the numbers of homozygous and compound heterozygous cases. The summary also highlights the specific combinations of pathogenic mutations observed in transfusion-dependent patients, thereby strengthening the link between molecular findings and clinical presentation.

3. **Proofreading and structure:**

The manuscript has been thoroughly proofread and edited to correct typographical errors to ensure consistent formatting throughout the text, including uniform font size and style. We are grateful for your constructive feedback, which has significantly improved the clarity and overall quality of our manuscript.

**Reviewer#3:**

We sincerely thank the Reviewer for her thoughtful and constructive comments, as well as for recognizing the value, structure, and rationale of our study. We greatly appreciate the time and effort taken to review our manuscript in such detail.

1. **Background:**

We have carefully revised and condensed this section to improve focus and flow, ensuring that only the most relevant information is retained to support the study rationale.

2. **Language and readability:**

The entire manuscript has been thoroughly edited for grammar, clarity, and sentence structure. We have also ensured that formatting is consistent throughout the text, including spacing around years, punctuation, and citation styles, and typographical errors in the references. We have reviewed the entire sections to ensure grammatical accuracy and improved readability.

3. **Tables and footnotes:**

As suggested, we have expanded the table footnotes to include full explanations of all abbreviations used. Additionally, how the previously reported pathogenic mutations have been described and cross-referenced to the relevant databases and guidelines for clarity and proper attribution.

4. **Figures of the sequencing chromatograms:**

Thank you for this excellent suggestion. We have now included sequencing chromatogram figures of the newly identified mutations to visually support our findings. We are very grateful for your detailed and constructive feedback, which has greatly helped us improve the quality, precision, and presentation of our manuscript.
